# Context-dependent effects of triiodothyronine on progesterone secretion and cAMP responses in rat ovarian tissues

**DOI:** 10.1530/EC-26-0464

**Published:** 2026-09-24

**Authors:** Chien-Chen Lu, Jou-Chun Chou, Hsi-Chang Shih, Shyi-Wu Wang, Paulus S Wang

**Affiliations:** ^1^ Department of Optometry, MacKay Junior College of Medicine, Nursing and Management, Taipei, Taiwan, R.O.C.; ^2^ Department of Medical Research, Shuang Ho Hospital, Taipei Medical University, New Taipei City, Taiwan, R.O.C.; ^3^ National Changhua Senior High School of Commerce, Changhua, Taiwan, R.O.C.; ^4^ Department of Physiology, School of Medicine, National Yang Ming Chiao Tung University, Taipei, Taiwan, R.O.C.; ^5^ Department of Physiology and Pharmacology, College of Medicine, Chang Gung University, Taoyuan, Taiwan, R.O.C.; ^6^ Medical Center of Aging Research, China Medical University Hospital, Taichung, Taiwan, R.O.C.

**Keywords:** triiodothyronine, progesterone, ovarian tissue, cAMP, gonadotropins, thyroid hormone

## Abstract

Thyroid hormones are essential regulators of metabolism and reproduction; however, their direct influence on ovarian steroidogenesis remains incompletely understood. This study examined the context-dependent effects of triiodothyronine (T3) on progesterone secretion and cyclic adenosine monophosphate (cAMP) signaling in rat ovarian tissues. Using *in vitro* static culture and *in vivo* thyroid status models, we assessed the impact of T3, thyroxine (T4), porcine follicle-stimulating hormone (pFSH), and human chorionic gonadotropin (hCG) across estrous phases. Progesterone and cAMP levels were quantified by radioimmunoassay (RIA). T3 significantly enhanced basal cAMP synthesis and progesterone secretion in non-luteal ovarian cells, particularly when administered prior to hCG stimulation. This priming effect was consistent across proestrus, estrus, and diestrus phases. Conversely, both T3 and T4 dose-dependently suppressed basal and hCG-induced progesterone secretion in luteal tissues and isolated luteal cells. *In vivo* experiments confirmed that hyperthyroid conditions elevated ovarian cAMP content, whereas hypothyroidism induced by propylthiouracil (PTU) sensitized gonadotropin responsiveness. Collectively, these findings demonstrate that T3 exerts dual and context-dependent effects on ovarian progesterone production, which are closely associated with modulation of cAMP signaling pathways. These results suggest that thyroid hormone status may influence ovarian function through cAMP-mediated mechanisms and highlight a potential link between thyroid dysfunction and altered reproductive endocrine regulation. Further studies are warranted to elucidate the underlying molecular pathways.

## Introduction

The ovary plays a central role in female reproductive physiology, not only by providing a hormonally balanced environment conducive to implantation but also by supporting early embryonic development until placental function is established. In the absence of fertilization, the corpus luteum undergoes luteolysis approximately 14 days post-ovulation, resulting in structural regression and the formation of the corpus albicans through infiltration by leukocytes, macrophages, and fibroblasts. Progesterone, a key steroid hormone synthesized by ovarian follicular and luteal cells, plays a crucial role in regulating the estrous cycle, maintaining pregnancy, and supporting lactation. During the follicular phase, estradiol predominates, whereas progesterone becomes the principal hormone during the luteal phase and gestation. Progesterone biosynthesis is rapidly initiated upon stimulation, converting intracellular cholesterol and intermediates into active hormone *via* enzymatic cascades. The hormone exerts its effects by binding to intracellular receptors, translocating to the nucleus, and modulating gene expression through mRNA transcription and protein synthesis (1, 2).

Thyroid hormones, including T4, T3, and reverse T3, are critical regulators of systemic metabolism and development (3, 4, 5). T4 serves as a prohormone for T3, which is 4–5 times more potent but less abundant and shorter-lived (6, 7). T3 acts *via* steroid-like mechanisms, entering target cells through passive diffusion or carrier-mediated transport, including monocarboxylate transporter 8 (MCT8) and organic anion-transporting polypeptide 1C1 (OATP1C1), binding to thyroid hormone receptor alpha (THRA) and thyroid hormone receptor beta (THRB), and influencing transcriptional activity (4, 8, 9, 10). Beyond genomic effects, T3 also modulates mitochondrial bioenergetics and non-genomic membrane-initiated signaling *via* integrin αvβ3 and phosphatidylinositol 3-kinase/protein kinase B (PI3K/Akt) pathways (11). Previous studies suggest that thyroid hormones may regulate calcitonin secretion through non-genomic mechanisms. In TT cells, a human medullary thyroid carcinoma cell line, T4 and its membrane-impermeable conjugate significantly increased calcitonin release, intracellular cyclic adenosine monophosphate (cAMP), and the activities of adenylyl cyclase (AC) and protein kinase A (PKA) without altering calcitonin mRNA. These effects were blocked by AC and PKA inhibitors, indicating that T4 stimulates calcitonin secretion *via* a cAMP-dependent PKA pathway (12).

Mitochondria play a central role in ovarian steroidogenesis, as the initial and rate-limiting steps of progesterone biosynthesis occur within this organelle (1). Following transport to the inner mitochondrial membrane, cholesterol is converted to pregnenolone by CYP11A1 (P450scc), initiating the steroidogenic pathway leading to progesterone production (1, 13). Enhanced mitochondrial function may support steroidogenic activity by increasing cellular energy availability and maintaining mitochondrial processes required for progesterone biosynthesis (11, 14). Given the established role of T3 in regulating mitochondrial bioenergetics, thyroid hormone signaling may contribute to ovarian steroidogenic function and progesterone production (14, 15, 16).

Clinical and experimental evidence robustly links thyroid dysfunction to gonadal impairment. Hypothyroidism is associated with diminished ovarian reserve, menstrual irregularities, reduced fertility, and ovarian atrophy, whereas hyperthyroidism may precipitate oligomenorrhea, luteal phase defects, and impaired lactation (5, 15, 17). In assisted reproductive technology (ART), thyroid autoimmunity and elevated thyroid-stimulating hormone (TSH) levels (>2.5 mIU/L) are associated with reduced oocyte yield, lower fertilization rates, and an increased risk of miscarriage (5, 18). Recent meta-analyses (19) confirm that subclinical hypothyroidism (SCH) is associated with lower antral follicle count (AFC) and anti-Müllerian hormone (AMH) levels. Furthermore, even high-normal TSH levels within the conventional euthyroid range may serve as a predictor for diminished ovarian reserve, particularly in women of advanced reproductive age.

Despite these advances, the cellular mechanisms by which thyroid hormones regulate ovarian steroidogenesis remain incompletely defined, particularly in non-luteal versus luteal compartments. Early work by Channing and Kammerman (20) revealed that T3, in combination with insulin, enhances luteinizing hormone (LH)- and follicle-stimulating hormone (FSH)-induced luteinization in porcine granulosa cells (20). More recent studies implicate insulin and insulin-like growth factor-1 (IGF-1) as selective stimulators of progesterone release in granulosa cells (21, 22). In this study, we investigated the direct effects of T3 on progesterone secretion in rat ovarian tissues, with a focus on non-luteal cells and the involvement of cAMP signaling. We examined several issues, including the following:

- the influence of gonadotropins on ovarian progesterone release,
- the stimulatory effect of T3 on progesterone secretion in rat ovaries,
- the impact of thyroid hormones on luteal tissue and cell progesterone output, and
- the role of T3 in modulating ovarian cAMP synthesis.

This study aimed to examine whether triiodothyronine modulates progesterone secretion in rat ovarian tissues under different hormonal contexts and whether such changes are accompanied by alterations in cAMP responses.

## Materials and methods

### Experimental design

#### Experiment I: effects of hCG and pFSH on ovarian progesterone secretion

Hormone treatments were designed to evaluate functional interactions between thyroid hormones and gonadotropins rather than sequential signaling events, with ‘basal’ conditions defined as ovarian tissues incubated without exogenous hormonal stimulation during the first incubation interval. Female rats were classified into three phases: proestrus, estrus, and diestrus. Ovaries were excised under ether anesthesia and cultured *ex vivo*. Each group was subdivided and treated with hCG at concentrations of 0, 0.05, 0.5, or 5 IU/mL for 60 min, followed by stimulation with pFSH at 0.1 μg/mL. In a parallel setup, ovaries were pretreated with pFSH at 0, 0.1, 1, or 10 μg/mL for 60 min, followed by hCG (0.5 IU/mL). Culture media were collected and extracted with diethyl ether for progesterone quantification *via* radioimmunoassay (RIA) (23).

#### Experiment II: effects of T3 on ovarian progesterone secretion across estrous phases

Female rats were classified into groups according to proestrus, estrus, and diestrus phases. Ovaries were cultured in Locke’s solution containing pFSH (0.1 μg/mL), T3 (1 nM), or both. After 60 min, media were replaced with fresh medium containing hCG (0.5 IU/mL) and incubated for an additional hour. Progesterone levels were measured by RIA (23).

#### Experiment III: effects of thyroid hormones on progesterone secretion in corpus luteum tissues

Corpora lutea were treated with T3 or T4 at concentrations of 10^−10^, 10^−8^, or 10^−6^ M, in the presence or absence of hCG (0.5 IU/mL). After 1 h of incubation, culture media were collected for progesterone quantification.

#### Experiment IV: dose and time effects of hCG on luteal cell progesterone secretion

Isolated luteal cells were cultured with hCG at 0, 0.05, 0.5, or 5 IU/mL. Media were collected at 0.5, 1, 2, and 4 h posttreatment. Progesterone concentrations were determined by RIA.

#### Experiment V: effects of T3 and T4 on luteal progesterone secretion

Luteinized ovarian tissues were obtained from immature rats primed with PMSG and hCG. Tissues were cultured in media containing T3 or T4 at 0, 10^−10^, 10^−8^, or 10^−6^ M, with or without hCG (0.5 IU/mL). After 60 min, media were collected for progesterone analysis.

#### Experiment VI: *in vivo* effects of thyroid status on ovarian cAMP synthesis

Female rats were divided into three groups: control (saline, 1 mL/kg), hypothyroid (propylthiouracil, PTU; 20 mg/kg), and hyperthyroid (T3, 5 μg/kg). Injections were administered subcutaneously once daily for 7 days. Ovaries were harvested and cultured with hCG (0.5 IU/mL) for 60 min. cAMP levels were measured using RIA.

#### Experiment VII: effects of thyroid hormones and gonadotropins on ovarian cAMP content

Rats in estrus and diestrus phases were identified by vaginal smear. Ovaries were cultured in Locke’s solution pretreated with 3-isobutyl-1-methylxanthine (IBMX) (1 mM) for 90 min. Ovarian tissues were subsequently incubated for 60 min in IBMX-containing medium supplemented with vehicle (control), T3 (1 or 10 nM), pFSH (0.1 μg/mL), or hCG (0.5 IU/mL). Tissues were then homogenized in 65% ice-cold ethanol and centrifuged. Protein content was determined by the Lowry assay (24), and cAMP levels in the resulting supernatant were quantified by RIA.

### Animals

Adult female Sprague–Dawley rats, weighing approximately 300 g, were procured from the Laboratory Animal Center of National Yang Ming University (approval number not applicable). The animals were housed under controlled environmental conditions, including a 14 h light:10 h darkness cycle (lights on from 06:00 to 20:00) and a temperature of 20–22°C. Food and water were provided *ad libitum*. All animal care and experimental procedures were performed in strict compliance with the ethical standards outlined in the Guide for the Care and Use of Laboratory Animals (NIH Publication No. 85-23, 1985 edition). All animal procedures complied with the institutional and national guidelines and were approved by the Institutional Animal Care and Use Committee (IACUC) of National Yang Ming University.

### Ovarian tissue culture

Rats were euthanized by decapitation, and ovaries were excised and cleared of surrounding connective tissue. Each ovary was placed in a 12 × 75 mm tube containing 1 mL Locke’s solution (23), oxygenated with 95% O_2_ and 5% CO_2_. After 90-min of pre-incubation at 37°C in a shaking water bath, the medium was replaced with 0.5 mL fresh medium containing hormones (hCG, T3, or FSH) or vehicle. The medium was collected every 60 min and replaced. Ovarian tissue was weighed post-culture, and progesterone levels in the medium were measured by RIA.

### Luteal tissue culture

Luteinized ovaries were obtained from hormonally primed rats (see below). Tissues were dissected, quartered, and cultured in 1 mL Locke’s solution under identical conditions as above. The medium was collected hourly and analyzed for progesterone.

### Induction of luteinization and luteal cell isolation

Immature female rats (22–28 days old) were primed with 50 IU pregnant mare serum gonadotropin (PMSG), followed 48–60 h later by 25 IU hCG to induce ovulation and luteinization. Six to seven days after hCG, rats were euthanized and ovaries harvested for luteal cell isolation.

Luteal cells were isolated using a modified protocol by Roess *et al.* (25, 26). The ovaries were minced and digested in a medium containing collagenase (1,500 U/g), BSA (200 mg/g), and hyaluronidase (0.25% w/v) under 95% O_2_/5% CO_2_ at 37°C with shaking. After sequential digestion and centrifugation (1,000 rpm (approximately 208 ***g***), 5 min, 20°C), the cells were filtered through a nylon mesh and resuspended in culture medium (DMEM/F12, 10% FBS, 2 mg/mL insulin, and antibiotics). Cell viability and count were assessed using trypan blue exclusion and hemacytometer. Luteal cell purity was not determined in this study using specific cellular markers. Cell isolation was performed according to the procedures of Chen *et al.* (25) and Roess *et al.* (26), which employ enzymatic dissociation of corpora lutea and have been widely used for studies of steroidogenic luteal cells.

### Luteal cell culture and hormone treatment

Viable luteal cells (1–2 × 10^5^ cells/well) were seeded into 24-well plates and incubated at 37°C in 5% CO_2_/95% O_2_ for 16–20 h. Hormonal treatments (T3, T4, and hCG) were administered in challenge medium (Medium 199 supplemented with glutamine, NaHCO_3_, HEPES, and BSA). After 1 h, media were collected for progesterone analysis (25).

### Culture medium extraction

Culture media were extracted with five volumes of diethyl ether using a vortex mixer (4010 Multi-Tube Vortexer, Corning, USA). After centrifugation (2,500 rpm (approximately 1,300 ***g***), 15 min, TJ-6, Beckman, USA), the organic phase was separated and dried under a fume hood. Samples were reconstituted in 0.1% gelatin–PBS for RIA.

### RIA for progesterone

Progesterone was measured using a previously validated RIA method (23). Standard progesterone was obtained from Calbiochem, and tritiated progesterone was obtained from Amersham. Sensitivity was 2 pg/tube; intra- and inter-assay CV values were 4.8 and 9.5%, respectively. Cross-reactivity was <8% for androgens and <0.3% for estrogens and corticosteroids.

### RIA for cAMP

cAMP was acetylated and measured using a previously described RIA method (27). Standards and tyr-cAMP were from Sigma; antiserum (CV-27 pool) was obtained from a previously established source (28); and ^125^I-cAMP was prepared *via* the chloramine-T method. Secondary antibody (anti-rabbit gamma globulin, ARGG) was obtained from a commercial or previously described source. Assay sensitivity ranged from 16 to 2,000 fmol.

### Statistical analysis

Data are expressed as mean ± SEM. Differences among groups were analyzed by two-way ANOVA. When the ANOVA indicated significant effects, group means were compared using Scheffé’s multiple comparison test. Student’s *t*-test was used for comparisons between two groups. Differences were considered significant at *P* < 0.05 and highly significant at *P* < 0.01.

## Results

### Dose-dependent effects of gonadotropins on ovarian progesterone secretion

In *ex vivo* rat ovarian cultures, hCG treatment did not produce a statistically significant dose-dependent increase in progesterone secretion across the estrous phases (Fig. 1A). In contrast, pFSH induced a robust, dose-dependent stimulation of progesterone release during both the estrus and diestrus phases (Fig. 1B). Furthermore, basal progesterone secretion levels were significantly higher during the diestrus phase than in the estrus phase (*P* < 0.01, Fig. 1C).

**Figure 1:**
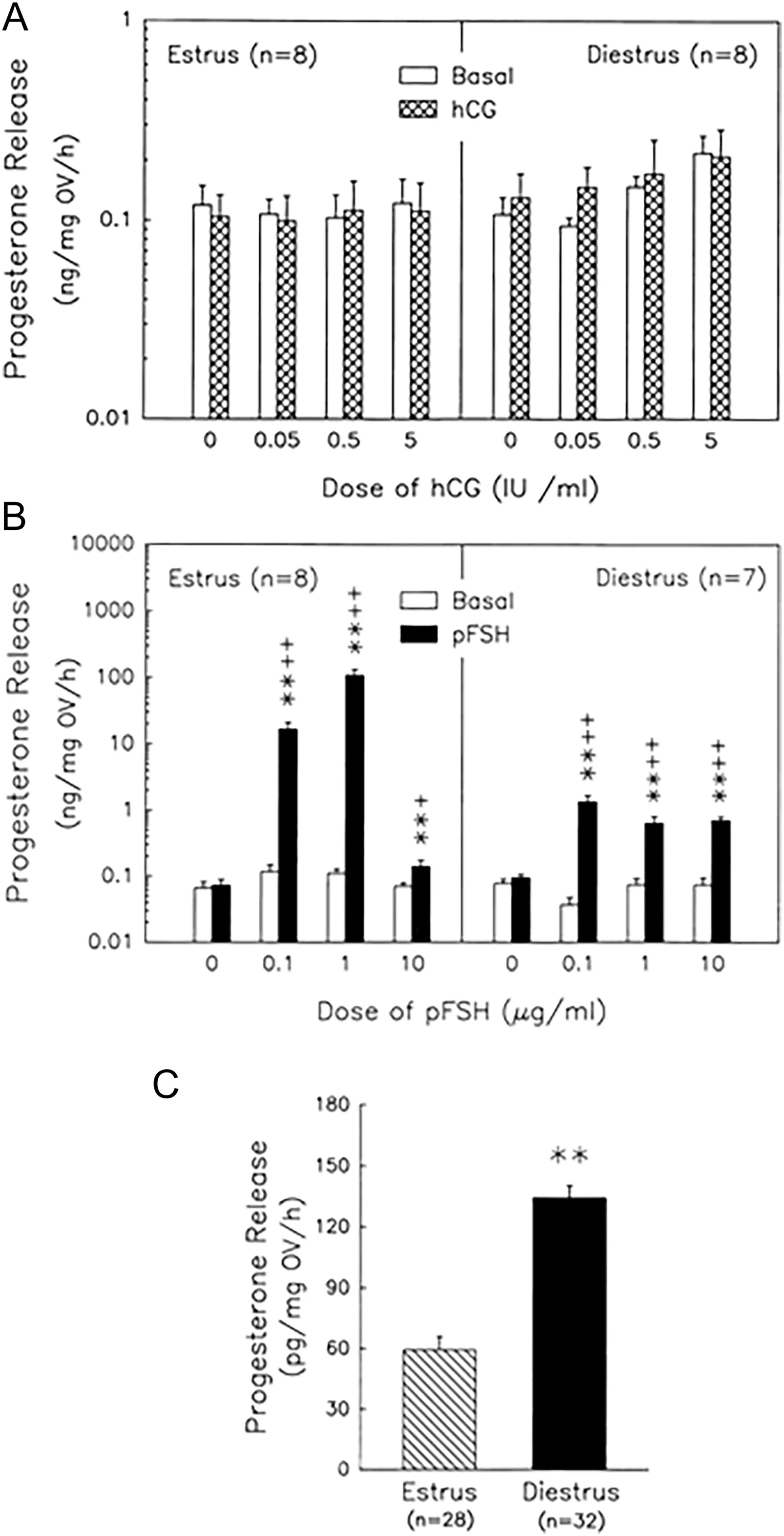
Effects of hCG and pFSH on ovarian progesterone release during the estrous cycle in rats. (A) Dose-dependent effect of hCG (0–5 IU/mL) on progesterone secretion from the ovaries of estrus and diestrus rats. Ovaries were excised and incubated in Locke’s medium (1 mL per flask) at 37°C. After 1 h of basal incubation, tissues were exposed to hCG for an additional one hour. Medium samples were collected, extracted with ether, and assayed for progesterone by RIA. (B) Effect of pFSH (0–10 μg/mL) under similar conditions. Statistical significance is indicated as *, ** for *P* < 0.05 and *P* < 0.01 versus the corresponding basal group and +, ++ for *P* < 0.05 and *P* < 0.01 versus pFSH = 0 μg/mL, respectively. (C) Basal progesterone release in estrus and diestrus rats. *P* < 0.01 compared with estrus rats.

### T3 as a priming factor for ovarian progesterone secretion

During the proestrus phase, pretreatment with T3 (1 nM), pFSH (0.1 μg/mL), or their combination significantly enhanced ovarian progesterone secretion, with subsequent hCG stimulation further amplifying this effect (Fig. 2A). In the estrus phase, all three pretreatment modalities significantly increased progesterone output, with T3 pretreatment followed by hCG stimulation yielding the most substantial response among all groups (Fig. 2B). For diestrus-phase ovaries, the combination of T3 and pFSH pretreatment significantly potentiated the stimulatory effect of hCG on progesterone secretion, indicating that T3 serves as a critical priming agent to enhance gonadotropin responsiveness (Fig. 2C).

**Figure 2:**
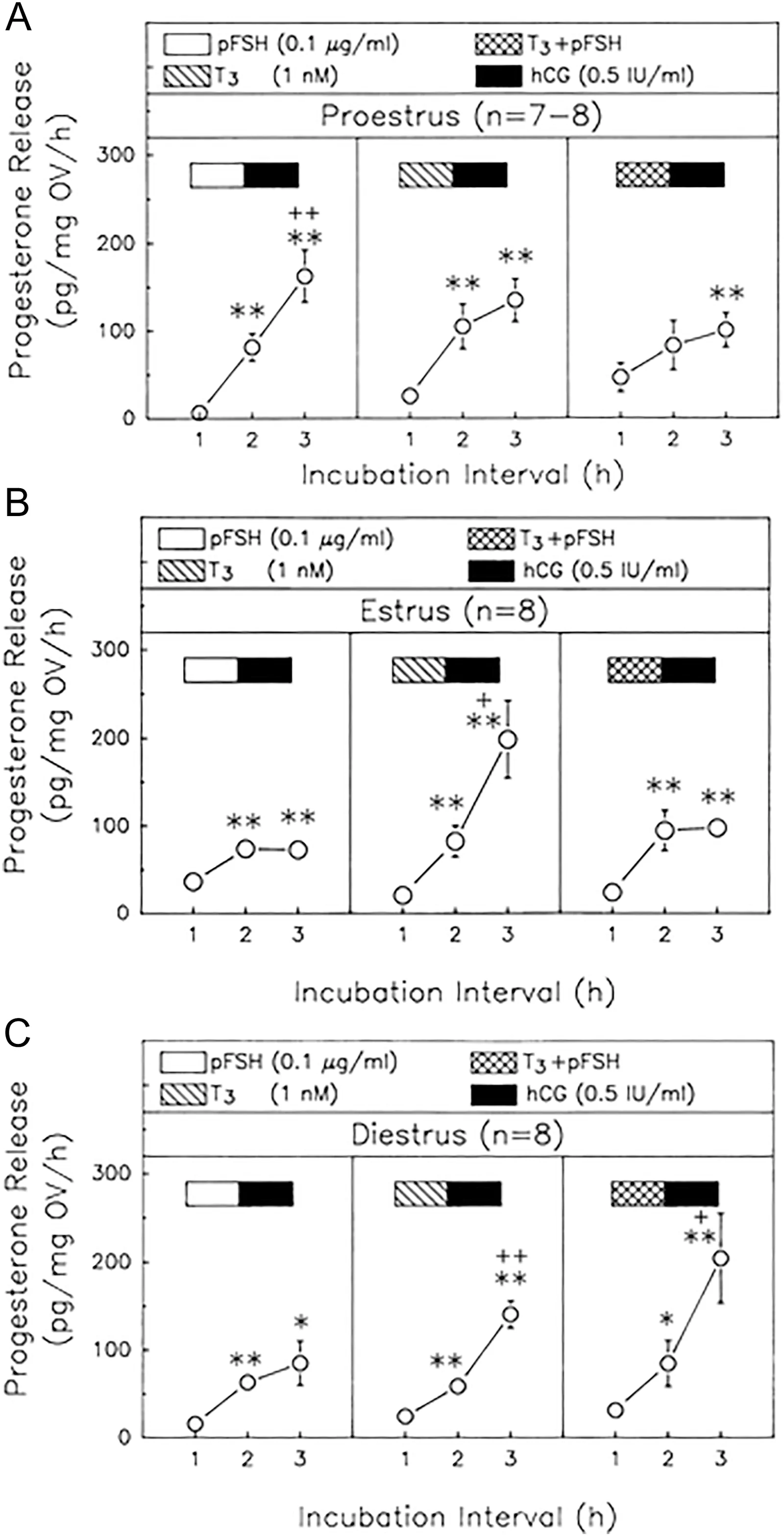
Time-dependent effects of pFSH and/or T3 on ovarian progesterone release prior to hCG stimulation in different stages of the estrous cycle. (A) Progesterone secretion in proestrus rats following pre-incubation with pFSH (0.1 μg/mL), T3 (1 nM), or T3 plus pFSH for 1 h before exposure to hCG (0.5 IU/mL). **P < 0.01 compared with the value at the first incubation interval; ++*P* < 0.01 compared with the value at the second incubation interval. (B) Progesterone secretion in estrus rats under the same experimental conditions. *P < 0.01 compared with the value at the first incubation interval; +*P* < 0.05 compared with the value at the second incubation interval. (C) Progesterone secretion in diestrus rats under the same experimental conditions. *, ** for *P* < 0.05 and *P* < 0.01 compared with the value at the first incubation interval; +, ++ for *P* < 0.05 and *P* < 0.01 compared with the value at the second incubation interval, respectively.

### Inhibitory actions of thyroid hormones on luteal function

In luteal tissues, T3 exerted a dose-dependent inhibitory effect on both basal and hCG-stimulated progesterone secretion, with significant suppression observed at a concentration of 10^−6^ M (Fig. 3A). T4 exhibited an even more potent inhibitory profile, significantly attenuating progesterone output in luteal tissues across all tested concentrations (10^−10^ to 10^−6^ M) (Fig. 3A). In isolated luteal cells, hCG treatment expectedly induced a significant dose- and time-dependent increase in progesterone secretion (Fig. 3B). However, T3 significantly inhibited this hCG-induced progesterone secretion in luteal cells at 10^−6^ M (Fig. 3C). Similarly, T4 dose-dependently suppressed progesterone output in isolated luteal cells, with marked inhibition of hCG-stimulated responses occurring at 10^−6^ M (Fig. 3D).

**Figure 3:**
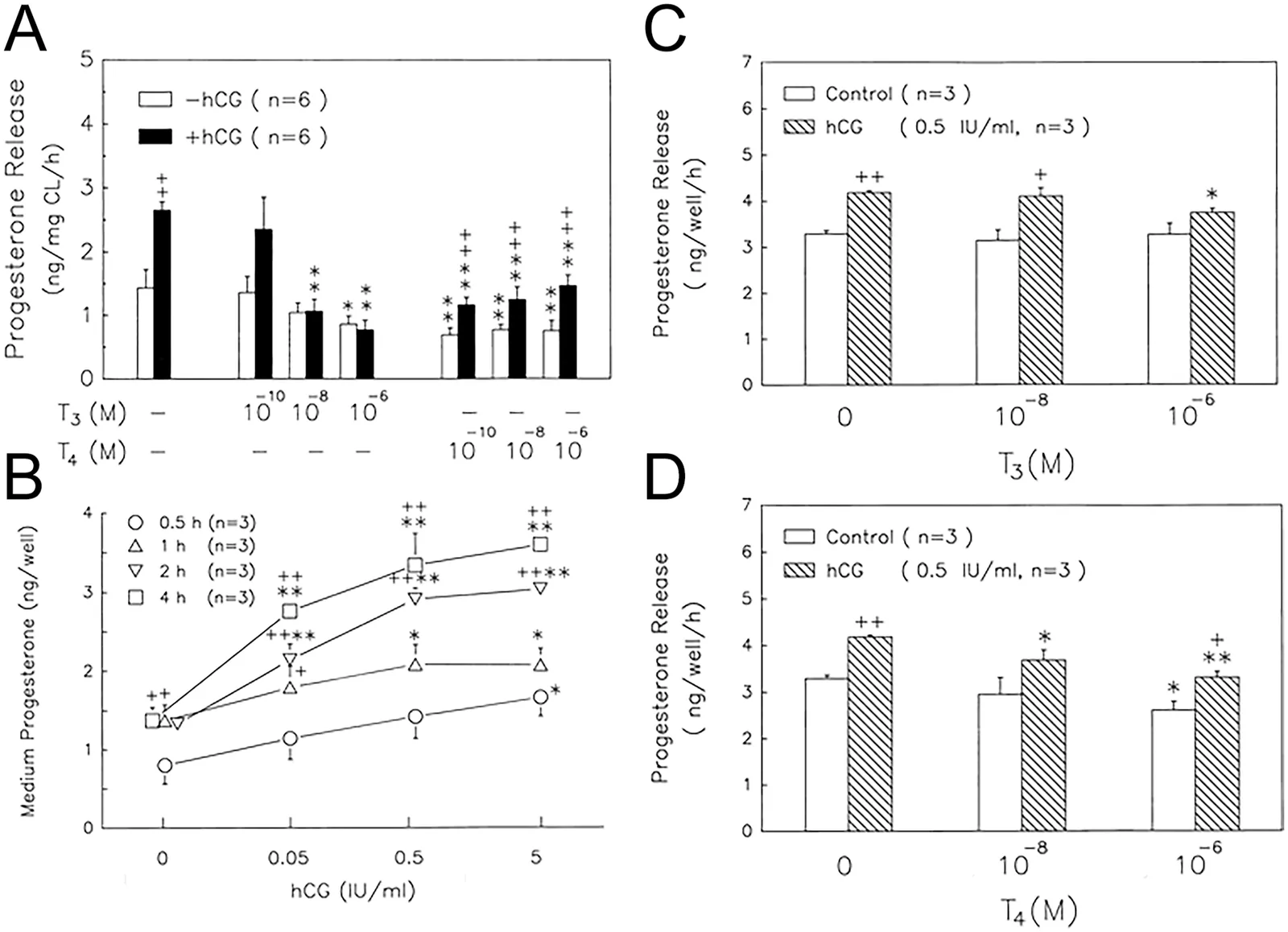
Effects of thyroid hormones and hCG on progesterone secretion from rat luteal tissue and isolated luteal cells. (A) Effects of T3 (10^−10^–10^−6^ M) and T4 (10^−10^–10^−6^ M) on progesterone release from corpus luteum tissue incubated with or without human chorionic gonadotropin (hCG; 0.5 IU/mL) at 37°C for 1 h. Statistical significance: *, ** indicate *P* < 0.05 and *P* < 0.01 versus the group treated with hCG alone; ++ indicates *P* < 0.01 versus the corresponding group without hCG. (B) Time course of progesterone secretion from isolated luteal cells in response to increasing doses of hCG (0, 0.05, 0.5, and 5 IU/mL). *, ** indicate *P* < 0.05 and *P* < 0.01 versus hCG = 0 IU/mL; +, ++ indicate *P* < 0.05 and *P* < 0.01 versus incubation time = 0.5 h. (C) Effect of T3 on basal and hCG-stimulated progesterone release from luteal cells. Corpora lutea were enzymatically dispersed using collagenase and hyaluronidase. Medium samples were collected and assayed by RIA. * indicates *P* < 0.05 versus T3 = 0 M; +, ++ indicate *P* < 0.05 and *P* < 0.01 versus control group, respectively. (D) Effect of T4 on basal and hCG-stimulated progesterone release from luteal cells under identical conditions. *, ** indicate *P* < 0.05 and *P* < 0.01 versus T4 = 0 M; +, ++ indicate *P* < 0.05 and *P* < 0.01 versus control group, respectively.

### Effects of thyroid status and hormonal stimulation on cAMP dynamics

*In vivo* thyroid status significantly modulated ovarian cAMP production: ovaries from T3-treated (hyperthyroid) rats exhibited higher basal cAMP levels compared with saline-treated and PTU-treated rats (Fig. 4A). While hCG stimulated cAMP production across all groups, the magnitude of the cAMP increase was most pronounced in the hypothyroid (PTU-treated) group, suggesting that chronic T3 deficiency may induce a sensitized or hyperresponsive state to gonadotropins (Fig. 4A). *In vitro* experiments further confirmed that T3 (10 nM) directly stimulates cAMP synthesis in estrus-phase ovaries, supporting an association between thyroid hormone exposure and changes in ovarian cAMP levels in non-luteal ovarian compartments (Fig. 4B).

**Figure 4:**
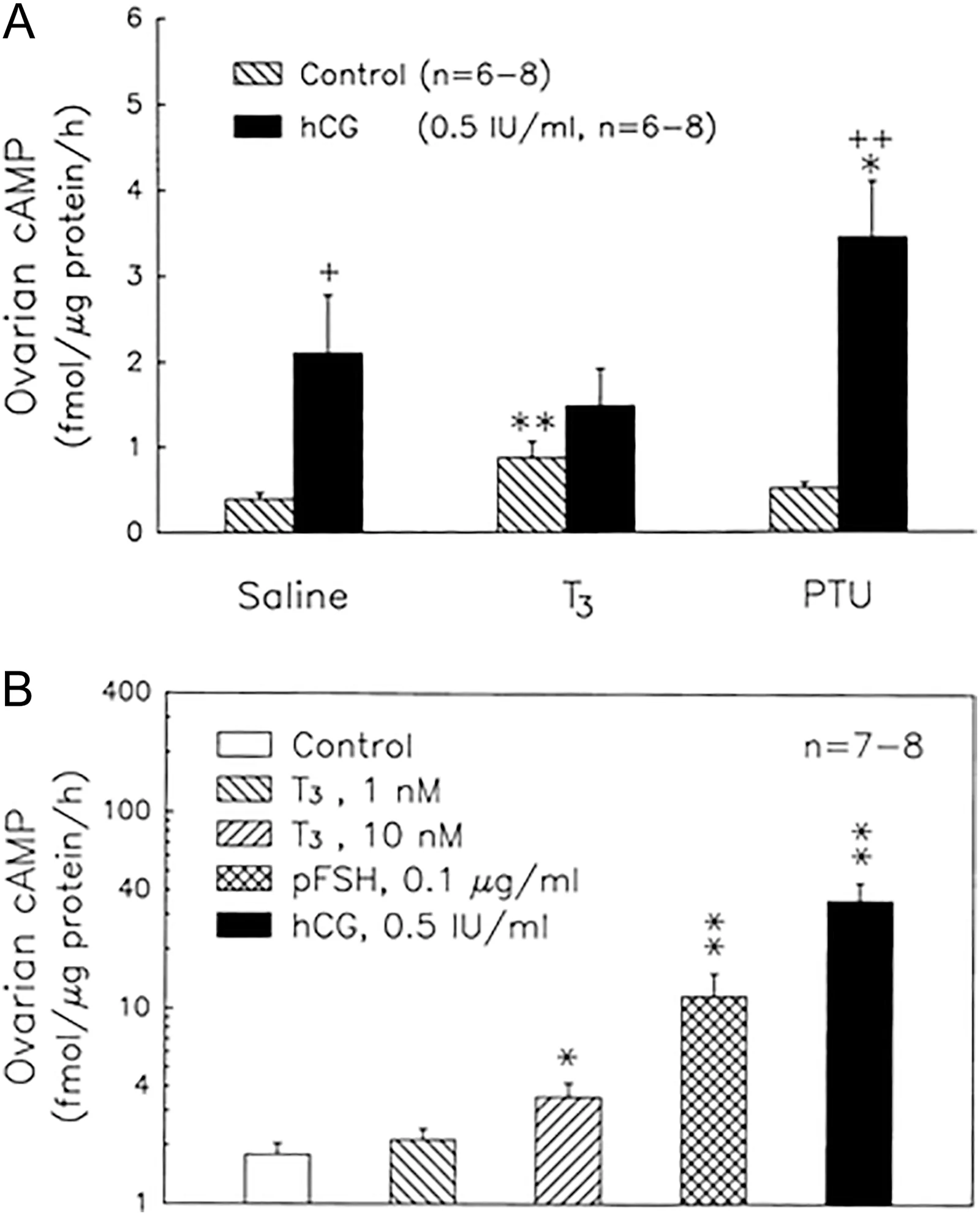
Effects of thyroid status and hormonal stimulation on ovarian cAMP production in rats. (A) *In vivo* effects of altered thyroid status on basal and human chorionic gonadotropin (hCG)-stimulated cyclic adenosine monophosphate (cAMP) production. Female rats received daily subcutaneous injections of saline (1 mL/kg, control), triiodothyronine (T3; 5 μg/kg, hyperthyroid), or propylthiouracil (PTU; 20 mg/kg, hypothyroid) for 7 days. Following the treatment period, excised ovaries were cultured *ex vivo* in the presence or absence of hCG (0.5 IU/mL) at 37°C for 60 min. Ovarian cAMP levels were quantified by radioimmunoassay (RIA) and normalized to total protein content. Statistical significance: *, ** indicate *P* < 0.05 and *P* < 0.01 versus the saline-treated group; +, ++ *P* < 0.05 and *P* < 0.01 versus the corresponding group without hCG. (B) *In vitro* effects of T3 and gonadotropins on ovarian cAMP accumulation. Ovaries harvested from estrus-phase rats were pre-incubated in Locke’s solution containing 1 mM IBMX for 90 min. Ovarian tissues were subsequently incubated for 60 min in IBMX-supplemented medium containing vehicle, T3 (1 or 10 nM), porcine follicle-stimulating hormone (pFSH; 0.1 μg/mL), or hCG (0.5 IU/mL). Tissues were homogenized in 65% ice-cold ethanol and centrifuged. The cAMP levels in the resulting supernatant were quantified by RIA and normalized to total protein content determined by the Lowry assay. Statistical significance: *, ** indicate *P* < 0.05 and *P* < 0.01 versus the vehicle-treated group, respectively.

## Discussion

The present study demonstrates that triiodothyronine (T3) modulates ovarian progesterone secretion in a context-dependent manner, with distinct effects observed in non-luteal versus luteal ovarian compartments. Using *ex vivo* ovarian tissue cultures, isolated luteal cells, and *in vivo* thyroid status models, we show that T3 and thyroxine (T4) are associated with alterations in progesterone output that parallel changes in ovarian cyclic adenosine monophosphate (cAMP) levels. The measurements obtained in this study do not distinguish between changes in cAMP production and degradation and therefore do not permit conclusions regarding the mechanism responsible for altered cAMP levels. These findings extend previous observations relating thyroid function to reproductive performance and provide functional insights into how thyroid hormones influence ovarian steroidogenic activity under different hormonal environments (11, 15). Both T3 and T4 suppressed progesterone secretion in luteal tissues and isolated luteal cells. Additional studies will be required to identify the mechanisms responsible for this effect.

The application of hCG in this research is supported by its recognized luteotropic activity and high receptor affinity, making it an established surrogate for LH (25, 29). pFSH, which promotes follicular development and progesterone synthesis, served as a comparative control (30). While pFSH elicited a robust dose-dependent increase in progesterone secretion, hCG alone showed limited efficacy in non-luteal tissue unless preceded by T3 or pFSH treatment. This observation suggests that hCG requires prior receptor priming or a synergistic hormonal context to exert its full effect on non-luteal ovarian cells. High luteinizing hormone/choriogonadotropin receptor (LHCGR) expression in luteinized cells typically facilitates strong LH/hCG-driven progesterone production, whereas non-luteinized granulosa cells require FSH priming to induce LHCGR expression and gain gonadotropin responsiveness (31, 32, 33). Differences in progesterone responses to pFSH and hCG may be related to receptor distribution within the non-luteal ovarian tissues examined in experiment I. During follicular development, granulosa cells express abundant FSH receptors, and FSH promotes steroidogenesis while inducing expression of the luteinizing hormone/choriogonadotropin receptor (LHCGR) (31, 33). By comparison, LHCGR expression is relatively low in non-luteinized granulosa cells before differentiation. As a result, hCG alone would be expected to have limited steroidogenic activity in heterogeneous non-luteal ovarian tissue. The greater response to pFSH is consistent with its established role in granulosa cell maturation and the development of LH/hCG responsiveness. T3 has been shown to further influence granulosa cell differentiation and enhance hCG-induced cAMP accumulation, indicating its role in establishing functional gonadotropin sensitivity (16, 34). Furthermore, while direct evidence linking T3 to receptor trafficking is emerging, LHCGR membrane availability is critically regulated by post-endocytic sorting and Rab11-associated recycling mechanisms (35).

Available evidence indicates that ovarian responses to thyroid hormones differ according to the cellular and physiological context. T3 has been reported to enhance gonadotropin-induced luteinization in porcine granulosa cells (20), whereas studies in human luteinized granulosa cells showed effects on hCG-stimulated progesterone secretion and cAMP accumulation (34). In addition, thyroid hormones are present in follicular fluid and have been associated with ovarian function in women undergoing assisted reproductive treatment (17). The ovarian response to thyroid hormones appears to differ according to cell type and functional status. In non-luteal ovarian tissues, T3 increased progesterone secretion and cAMP accumulation. In contrast, both T3 and T4 reduced progesterone production in luteal tissues and isolated luteal cells. Similar variability has been reported in previous studies of granulosa and luteal cells. The current data show divergent effects in non-luteal and luteal tissues, further emphasizing the importance of cellular context in ovarian hormone regulation.

The effects of T3 are not restricted to cAMP signaling. Thyroid hormones are transported into cells by membrane carriers such as MCT8 and act through thyroid hormone receptors THRA and THRB. Receptor-mediated signaling regulates genes involved in cellular differentiation, metabolism, and steroidogenesis (4, 8, 10, 36). Non-genomic actions of T3 include activation of PI3K/Akt signaling (11, 14). This pathway has recognized roles in cellular metabolism, mitochondrial function, and steroidogenesis. Its involvement in the enhanced response to hCG observed after T3 treatment warrants further investigation.

Phase-specific variations in progesterone production align with classic reports detailing fluctuations in circulating hormones throughout the rat estrous cycle (37). Higher basal progesterone levels during diestrus likely reflect phase-specific changes in ovarian cellular composition and gonadotropin receptor availability. In line with the priming effects described above, T3-associated enhancement of progesterone secretion was observed across estrous phases, supporting a role for thyroid hormones in maintaining ovarian steroidogenic competence. This is consistent with human clinical data showing that T3 enhances hCG-induced cAMP and progesterone production in luteinized granulosa cells (34). Notably, hypothyroid conditions induced by PTU sensitized ovarian tissue to hCG stimulation, suggesting a compensatory mechanism that may have clinical relevance for managing thyroid-related infertility (5, 38). Mitochondrial function is an important determinant of steroid hormone production because several key steroidogenic reactions occur within this organelle. Enhanced mitochondrial activity may increase the availability of energy required for cholesterol transport and pregnenolone formation, two critical steps in progesterone biosynthesis (1, 13, 14). The increase in progesterone secretion observed in non-luteal ovarian tissues after T3 treatment is compatible with a role for mitochondrial regulation in ovarian steroidogenesis.

Consistent with the functional associations described above, alterations in thyroid status were accompanied by corresponding changes in ovarian cAMP responsiveness, a central regulator of steroidogenesis (13, 39). The present data demonstrate differences in ovarian cAMP accumulation following thyroid hormone treatment, but they do not identify the mechanism responsible for these changes. Changes in thyroid status were accompanied by corresponding changes in ovarian cAMP accumulation and responsiveness to gonadotropin stimulation (40). The contribution of adenylate cyclase, phosphodiesterase, or other signaling pathways to these responses could not be determined from the present data. T3 treatment increased basal ovarian cAMP accumulation, whereas ovaries from PTU-treated animals exhibited a greater cAMP response to hCG stimulation. This pattern may reflect altered ovarian sensitivity to gonadotropin stimulation under hypothyroid conditions. In contrast, both T3 and T4 reduced progesterone secretion in luteal tissues and isolated luteal cells (15, 40). T4 inhibited progesterone secretion over a wider concentration range than T3. In addition to serving as a precursor for intracellular T3 production, T4 has been reported to exert biological effects through non-genomic pathways. The relative roles of T3 and T4 in the regulation of luteal function remain incompletely defined. The results obtained in this study indicate different responses to thyroid hormones in luteal and non-luteal ovarian tissues. Figure 5 is intended as a schematic interpretation of the present findings. PKA signaling, StAR function, mitochondrial cholesterol transport, and LHCGR-mediated gonadotropin responsiveness shown in the figure are supported by previous studies (1, 13, 29, 32, 35) and were not examined directly in this study. The data obtained in this study do not identify the mechanism responsible for the changes in cAMP accumulation. Consequently, the scheme shown in Fig. 5 should be viewed as a conceptual interpretation of the findings rather than a validated signaling pathway.

**Figure 5:**
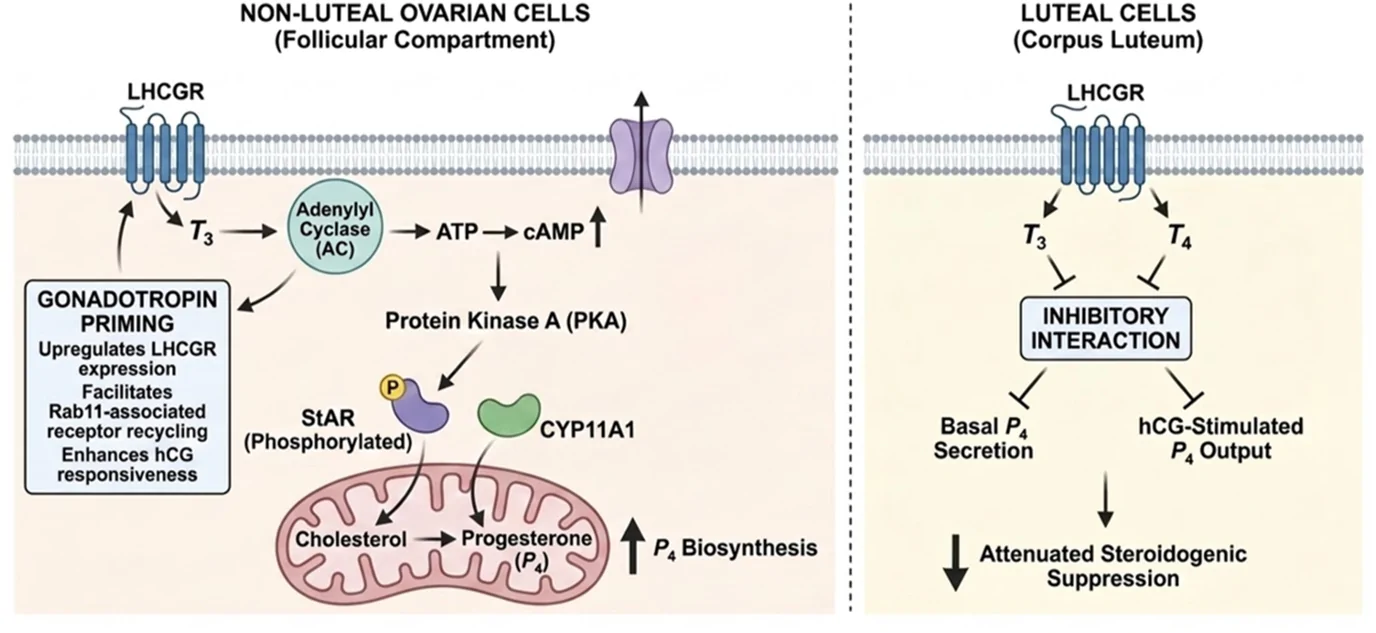
Conceptual overview of context-dependent associations between triiodothyronine, cAMP response, and progesterone secretion in rat ovarian tissues. T3 increased cAMP accumulation in non-luteal ovarian tissues. PKA signaling, StAR function, mitochondrial cholesterol transport, and LHCGR-mediated gonadotropin responsiveness shown in the figure are derived from previous studies (1, 13, 29, 32, 35) and were not evaluated in the present experiments. This figure is presented as a schematic summary of the findings. Both T3 and T4 reduced progesterone secretion in luteal tissues and isolated luteal cells, demonstrating differential ovarian responses to thyroid hormones.

Regulation of female reproduction by thyroid hormones is not limited to the ovary but also involves the hypothalamic–pituitary–gonadal (HPG) axis. Altered thyroid status affects gonadotropin secretion, ovarian cyclicity, and fertility (5, 16, 37, 39). The ovarian effects observed in this study should therefore be considered within the broader context of endocrine regulation of reproduction. Interactions between thyroid and reproductive function may contribute to the reproductive abnormalities reported in both hypothyroid and hyperthyroid conditions. From a translational perspective, these findings carry significant implications for ART and endocrine disorders, such as polycystic ovary syndrome and premature ovarian insufficiency. Understanding how thyroid hormones modulate gonadotropin sensitivity could inform strategies to optimize ovarian stimulation protocols and improve fertility outcomes in women with thyroid dysfunction (16, 38).

### Study limitations

Several limitations of the present study should be acknowledged. First, ovarian tissues used in these experiments represent heterogeneous cell populations, particularly within the non-luteal compartment, and thus, the observed hormonal effects cannot be attributed to specific ovarian cell types. Second, changes in cAMP levels and progesterone secretion occurred concurrently, but the signaling mechanisms responsible for these responses were not examined. Measurements of adenylate cyclase activity, phosphodiesterase activity, and other regulators of cAMP turnover were not included in the experimental design. As a result, the observed differences in cAMP accumulation cannot be attributed specifically to changes in cAMP production or degradation. Luteal cell purity was also not determined using cell-specific markers. Because the cell isolation procedure relied on enzymatic dispersion, contamination by other ovarian cell types cannot be ruled out. Consequently, conclusions regarding intracellular signaling pathways should be interpreted cautiously. Future studies employing defined ovarian cell populations and molecular analyses will be necessary to clarify the precise mechanisms underlying thyroid hormone-mediated modulation of ovarian steroidogenesis.

## Conclusion

In summary, triiodothyronine modulates ovarian progesterone secretion through context-dependent mechanisms that are closely associated with alterations in cAMP signaling. These findings highlight a functional link between thyroid hormone status and ovarian endocrine regulation and suggest that thyroid hormones may play an important role in modulating gonadotropin responsiveness and reproductive outcomes (5, 38).

## Declaration of interest

The authors declare that there is no conflict of interest that could be perceived as prejudicing the impartiality of the research reported.

## Funding

This work was supported by the National Science Council, Taiwan, Republic of China (grant number NSC85-2231-B010-053).

## Author contribution statement

CCL conceived the study; performed formal analysis and investigation; and wrote, reviewed, and edited the manuscript. JCC reviewed and edited the manuscript. HCS carried out investigation and formal analysis. SWW contributed resources. PSW conceived the study, designed the methodology, acquired funding, and reviewed and edited the manuscript.
